# First-Principles Path Integral Monte Carlo Studies of the Pseudo Jahn–Teller Effect in the Aromatic Cyclo[10]carbon

**DOI:** 10.1021/acs.jpca.4c08620

**Published:** 2025-02-24

**Authors:** Anna H. James, Martina Kaledin, Alexey L. Kaledin

**Affiliations:** † Department of Chemistry & Biochemistry, Kennesaw State University, 370 Paulding Ave NW, Box # 1203, Kennesaw, Georgia 30144, United States; ‡ Cherry L. Emerson Center for Scientific Computation and Department of Chemistry, Emory University, 1515 Dickey Drive, Atlanta, Georgia 30322, United States

## Abstract

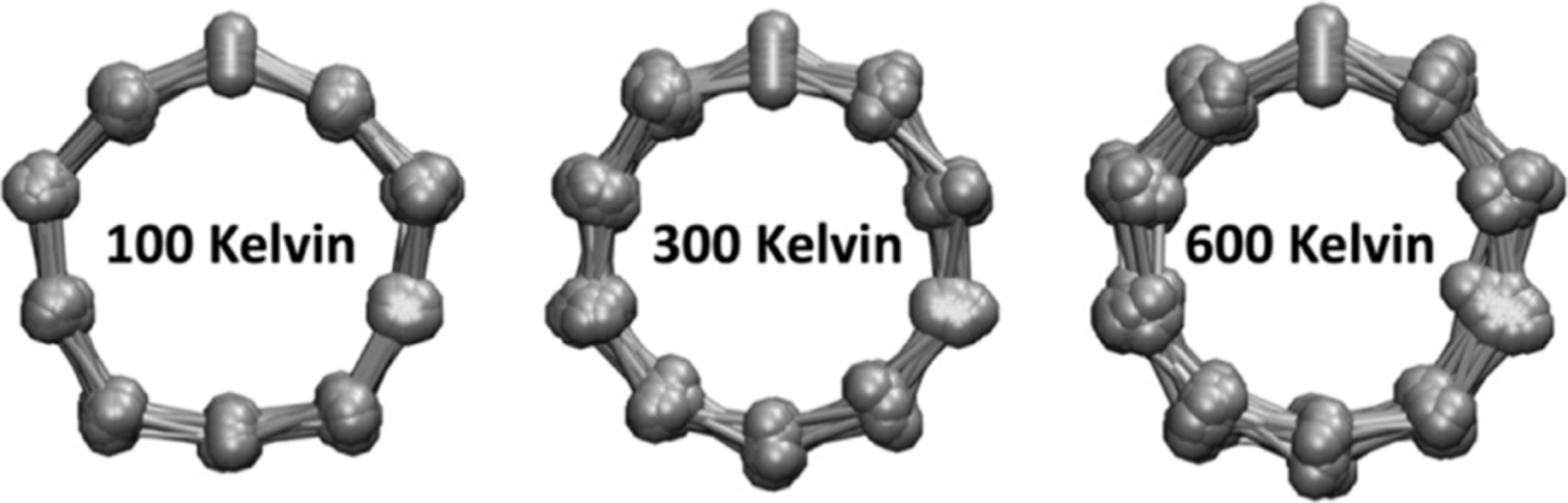

There has been renewed interest in carbon nanoscale structures. Experimental measurements at 4.7 K and subsequent first-principles-based vibrational diffusion Monte Carlo simulations at 0 K recently showed that the aromatic cyclo[10]carbon prefers a *D*_5*h*_ pentagon-like structure to a regular *D*_10*h*_ decagon. This symmetry breaking is due to the second-order Jahn–Teller effect (JTE) and has been amply described in the literature for the cumulenic cyclo[4*m* + 2]carbon clusters. Yet temperature dependence of the JTE in cyclo[4*m* + 2]carbon clusters in general and the cyclo[10]carbon in particular has not been studied systematically. In this work, we employ path integral Monte Carlo simulations on a first-principles-derived permutationally invariant potential energy surface (PES) to examine the JTE in cyclo[10]carbon as a function of temperature. The PES was trained on a set of τHCTH/cc-pVQZ energies sampled up to ∼7.7 eV above the *D*_5*h*_ global minimum and locally adjusted to a high-level benchmark (reported by others) of the 812 cm^–1^ electronic energy difference between the *D*_5*h*_ global minimum and the *D*_10*h*_ transition state. The calculations show a strong JTE at lower temperatures with a dominant *D*_5*h*_ composition at 100 K and a gradually diminishing JTE at higher temperatures with a washed-out pentagonal structure above 300 K.

## 1 Introduction

Carbon clusters (C_*n*_), which consist of only two-coordinated atoms, have been the subject of intensive research due to their strong reactivity and facile isomerization.^1−6^ sp-hybridization in carbon clusters allows for an in-plane π electron system and an out-of-plane π electron system, while sp^2^ hybridized carbon clusters have a single π-electron system.^3^ Even-numbered carbon clusters have been isolated experimentally in cyclic forms in the singlet electronic state and linear forms in the triplet electronic state.^7,8^ Chemical properties of the even-numbered cyclo[*n*]carbons have been shown to vary with size depending on whether *n* is a multiple of four, i.e., *n* = 4*m*, or a multiple of two of the form *n* = 4*m* + 2, for any integer *m*.^6^ More generally, the structure of a cyclo[4*m* + 2]carbon is predicted to be cumulenic with a nonalternating =C=C=C= double bond pattern, while that of a cyclo[4*m*]carbon is expected to be polyynic (acetylenic) with a well-demarcated ≡C–C≡C– bond alteration pattern.^9−11^

Interpretation of the stability of the even-numbered cyclo[*n*]carbons has been offered in terms of aromaticity, antiaromaticity, double aromaticity, double antiaromaticity, the Jahn–Teller effect (JTE), and the second-order JTE, i.e., Peierls effects.^3,12−19^ Aromaticity plays a critical role in chemical reactions by stabilizing the transition state or product by delocalizing π-electrons within aromatic systems. Conversely, antiaromaticity can destabilize reactants by disrupting electronic conjugation.^20^ According to Hückel’s rules, a molecule is considered aromatic if it contains 4*m* + 2 π-electrons within a conjugated cyclic system. Computational methodologies are frequently employed to analyze and predict the properties of transition states in aromatic and antiaromatic cyclic structures, providing valuable insights into their reactivity and stability. The symmetry-breaking phenomena witnessed in aromatic cyclo[*n*]carbons is theorized to be an effect of aromaticity or a second-order Jahn–Teller distortion.^3^

The cyclo[10]carbon stands out as particularly intriguing, if only from a computational standpoint because it satisfies Hückel’s rule for aromaticity,^21^ which imparts extra stability to its decagonally symmetric cyclic conformation while competing with the JTE, and possesses an unusually complicated electronic structure.^7,22−24^ Until the recent measurements of Sun et al.^25^ which described the cyclo[10]carbon as having a cumulenic pentagonal structure with a *D*_5*h*_ symmetry at 4.7 K, there was no strong consensus in the computational community on whether in experimental conditions it is a regular decagon (*D*_10*h*_) or a distorted pentagon with a *D*_5*h*_ symmetry. A vast majority of all the reported electronic structure calculations have correctly predicted a cumulenic *D*_5*h*_ structure as the lowest energy stationary point on the global potential energy surface (PES), yet estimates of the *D*_10*h*_ barrier height and the zero-point vibrational energy corrections have sometimes produced unstable or extremely weakly stable *D*_5*h*_ structure, leading to speculation of a vibrationally averaged *D*_10*h*_ structure at 0 K.^22−24,26,27^ For instance, the best estimate for the enthalpy barrier, based on harmonic analysis, to the *D*_5*h*_–*D*_10*h*_–*D*_5*h*_ isomerization at 0 K, ∼0.1 kcal/mol, was reported by Karton and Thimmakondu^27^ using an all-electron CCSDT(Q)/CBS treatment. Their calculation essentially reaffirmed cyclo[10]carbon as having a vibrationally averaged *D*_10*h*_ structure as had been presumed in previous decades. However, in an effort to elucidate this issue with an exact adiabatic treatment of nuclear motion, a vibrational diffusion Monte Carlo (DMC) simulation of the C_10_ cluster in full dimensions using an *ab initio* derived PES fully reflected the experimental findings and showed that at 0 K the C_10_ cluster is, in fact, a distorted and rather strongly localized pentagonal cumulene possessing of a *D*_5*h*_ symmetry.^28^

Temperature effects in the vibrational structure of cyclo[10]carbon have occasionally been brought up over the years in a speculative manner.^22,26^ Early, and to our knowledge, the only attempts to examine the effect of temperature on the C_10_ cluster stability were carried out by Andreoni et al. using classical MD simulations based on a DFT with the local density approximation.^29^ Their calculations revealed a stable *D*_5*h*_ structure up to 70 K which became floppy by 200 K and oscillated between the two identical pentagon-like *D*_5*h*_ minima while traversing the *D*_10*h*_ inversion point with an oscillation frequency equivalent to ∼2000 cm^–1^. The authors reported a *D*_5*h*_–*D*_10*h*_ inversion barrier height to be 806 cm^–1^, remarkably (and likely fortuitously) close to the CCSDT(Q)/CBS benchmark of 812 cm^–1^ derived by Karton and Thimmakondu.^27^ Assuming an uncoupled one-dimensional reaction coordinate and no tunneling, this barrier height predicts the *D*_5*h*_ structure to be stable at least up to 1100 K. To this end, 1-D quantum vibrational calculations on a PES fitted in part to the CCSDT(Q)/CBS benchmark appear to support the assumption of a high-temperature ceiling for the *D*_5*h*_ structure stability of the *D*_5*h*_ over *D*_10*h*_ configuration up to ∼1000 K.^28^ Nevertheless, the projected temperature ceiling could be overestimated due to the fact that a 1-D quantum treatment does not account for the degeneracies in the *D*_5*h*_ and *D*_10*h*_ point groups, the resultant mode coupling effects, and possibly the degree of the out-of-plane vibrational interference with the Jahn–Teller coordinate.

In the present work, we examine the JTE in cyclo[10]carbon quantum mechanically in full dimensionality as a function of temperature. We note that the well-documented *D*_10*h*_ symmetry breaking in cyclo[10]carbon is a routine manifestation of the JTE, and more specifically of the pseudo (or the second order) JTE since the ground singlet electronic state, S0, in the *D*_10*h*_ configuration is nondegenerate, and the first excited singlet S1 is found to be ∼3.5 eV higher (see the Supporting Information). This leads to the formation of a symmetric double-well energy profile, where the unstable high symmetry configuration (*D*_10*h*_) acts as a transition state point connecting two (or more) stable identical lower symmetry configurations (*D*_5*h*_). For a more comprehensive take on the JTE, the reader is referred to the comprehensive review of Bersuker.^30^ Symmetry-breaking of the *D*_10*h*_ configuration is expected to originate from electronic energy stabilization due to the in-plane and out-of-plane π-orbital resonances.^15,17^

Given the high dimensionality of the system, Monte Carlo approaches are most suitable, as previously demonstrated for the 0 K simulations with DMC.^28^ Since nonzero temperature simulations in DMC require a highly specialized treatment of nodal structure of the wave function,^31^ we employ Feynman’s path integral approach^32^ to the quantum vibrational problem with Metropolis Monte Carlo sampling of the configuration space.^33−36^ We extend the previously published PES for the 0 K DMC simulations (**PES_A**) to be suitable for temperatures up to 600 K and test it for much higher energy regions resulting in a new PES version, heretofore labeled as **PES_B1**.

## 2 Computational Methods

### 2.1 PES Definition and Its Refinement

Here, we briefly describe the analytic form used in the learning of the PES. Our approach is to take the one-electron expression for the Hartree–Fock energy augmented with a correction term and the nuclear repulsion energy^37^
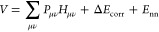
1and use it as a fitting function for PES. This approach was first introduced and validated by us for a number of nontrivial molecules where we showed an order of magnitude improvement in fitted RMSEs compared with conventional PES fitting methods.^37,38^ In eq 1, *P*_μν_ are the bond-order charge density matrix elements; *H*_μν_ are the elements of the core-Hamiltonian; Δ*E*_corr_ is the correlation energy acting as a correction, and *E*_nn_ is the nuclear repulsion energy. The slowly varying density matrix elements and the correlation energy are easier to represent analytically than the full potential energy on the LHS of eq 1 and can be done so with substantially fewer polynomial terms.^37,38^ Thus, each of the elements *P*_μν_ and the standalone term Δ*E*_corr_ are expressed as linear combination of permutation-invariant polynomials PIPs.^39^

**Figure 1:**
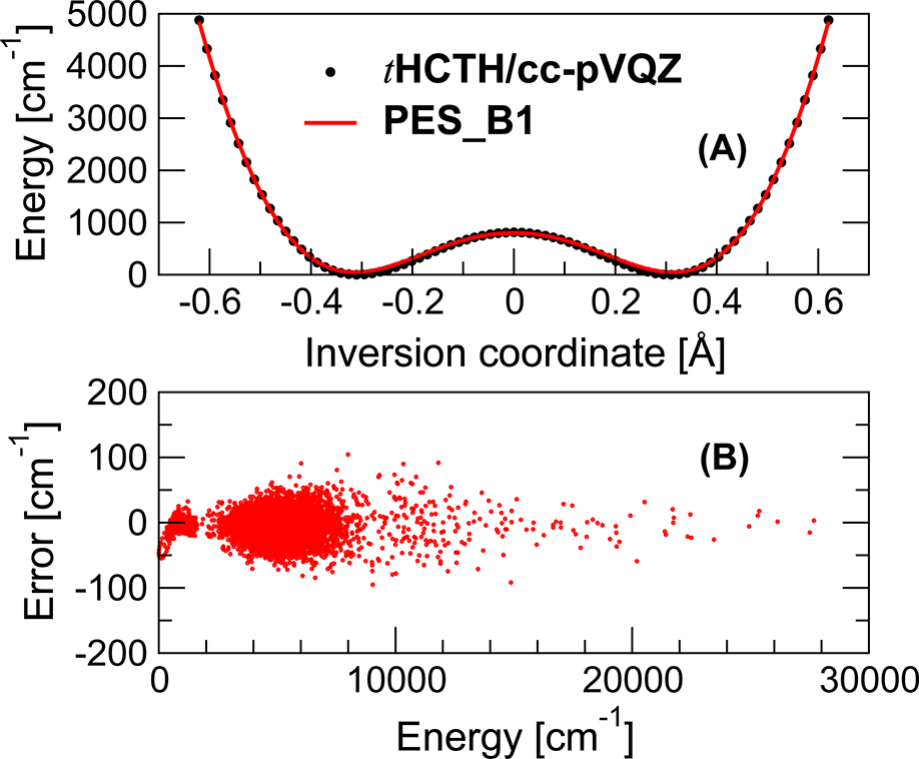
(A) Illustration of the *D*_5*h*_–*D*_10*h*_–*D*_5*h*_′ inversion reaction coordinate with the τHCTH/cc-pVQZ training data (see the text), morphed to reproduce the 812 cm^–1^ benchmark barrier height, (black dots) and the fitted **PES_B1**, (red line). The two *D*_5*h*_ minima are at ±0.37 Å, and the inversion transition state *D*_10*h*_ is at 0 Å. The fitted **PES_B1** barrier height is 756 cm^–1^. The RMSE of the symmetric 1-D cut with 21 unique data points is 29 cm^–1^. (B) τHCTH/cc-pVQZ training set of 5637 configurations and the error distribution of **PES_B1** as a function of the energy above the *D*_5*h*_ global minimum. We note the tendency of the fit to do well in the high energy region, suggesting **PES_B1** stability beyond 600 K. The RMSE of the training set is 22 cm^–1^.

The first version of the cyclo[10]carbon PES,^28^ named **PES_A**, was trained on τHCTH^40^/cc-pVQZ data which were morphed in the vicinity of the *D*_5*h*_ global minimum to reproduce the 812 cm^–1^ CCSDT(Q)/CBS *D*_5*h*_–*D*_10*h*_ barrier benchmark.^27^ The morphing procedure is described in detail in our preceding publication (ref (28)) where we also justified our use of the τHCTH/cc-pVQZ level of theory for mapping out the PES. Briefly, we showed that of several tested pure and hybrid density functionals and correlation consistent basis sets, the closest result for the benchmarked barrier height was achieved with τHCTH/cc-pVQZ which yielded a value of 742 cm^–1^. We previously stress-tested **PES_A** by means of extensive DMC simulations with ∼10^7^ sampled configurations.^28^ It was found that the surface was reliable at energies of up to ∼2 eV above the global minimum. Present exploratory path integral Monte Carlo (PIMC) simulations (to be outlined below) at lower temperatures using **PES_A** have initially shown a robust behavior by the surface. It was only when the temperature reached 600 K that we observed occasional energy collapse events caused by sampling of chemically unrealistic configurations. Further simulations at 800 and 1600 K exposed additional unstable regions, and therefore, we added 92 high-energy configurations to the training set, mostly corresponding to large amplitude out-of-plane deformations. To simultaneously improve PES accuracy near the key stationary points, we added 41 configurations corresponding to the *D*_5*h*_–*D*_10*h*_ isomerization coordinate (cf. Figure 1). The present PES was refitted to a total of 5637 τHCTH/cc-pVQZ morphed data, resulting in the current version which we presently label **PES_B1**. Remarkably, despite the inclusion of the high-energy configurations, with the energies reaching up to ∼7.7 eV above the global minimum, the unweighted training set errors are contained within ∼100 cm^–1^, and the cumulative RMSE is 22 cm^–1^, as shown in Figure 1. This PES was extensively retested at 600 K without exhibiting events of energy collapse. Simulations at 800 K, however, still showed occasional collapse events, but nonetheless, sufficient numbers of configurations could be generated to derive average properties. Our present conclusion is that **PES_B1** is expected to be fully useable for quantum simulations up to 600 K and partially useable up to 800 K.

### 2.2 Details of the PIMC Procedure

Properties are evaluated as quantum mechanical averages of the corresponding operator  weighted by the Boltzmann operator
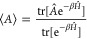
2where β = 1/*kT* is the inverse temperature and *H* is the Hamiltonian. In Feynman’s path integral formulation, the canonical partition function  is written as a multidimensional integral
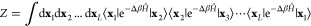
3where **x**_*i*_ is a 3*N* dimensional vector of atomic Cartesian coordinates (for *N* atoms in their center of mass frame) and Δβ = β/*L*. For large enough *L*, the matrix elements in eq 3 are calculated using the high-temperature approximation^34,41^
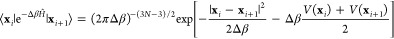
4assuming the atomic units and mass weighted Cartesians. Then, the path integral expression for the partition function becomes
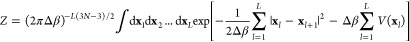
5with the periodic condition **x**_*L*+1_ = **x**_1_, and we rewrite the Boltzmann density as
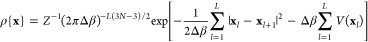
6

The property in eq 2 is evaluated as a Boltzmann-weighted integral of the path-averaged operator^35^
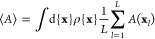
7using standard Metropolis Monte Carlo algorithms.^33^ As our primary goal is to investigate the temperature effect on the average structure, we calculate two relevant properties: (i) the radial distribution function (RDF) of the internuclear distances
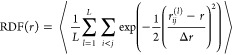
8where first sum runs over the *L* path slices and the second sum runs over all nucleus-pairs *i* and *j* with distance *r*_*ij*_^(*l*)^ apart and Δ*r* is the binning parameter, and (ii) the rovibrational enthalpy, i.e., the internal energy *U*(35)
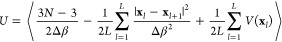
9

Additional details are provided in the Supporting Information.

To carry out the numerical simulations in a systematic manner, we make use of the quantum control factor Δβω* where ω* is the characteristic frequency, typically describing a fast vibrational mode. For a chosen ω*, the quantum control factor determines the accuracy of path integration, and in the limit of Δβω* ≪ 1, it leads to exact quantum results. Presently, we take ω* as the highest fundamental frequency of the global minimum *D*_5*h*_, 2025 cm^–1^ at the τHCTH/cc-pVQZ level of theory, corresponding to a collective C=C stretch, more specifically to the cumulene/acetylene =C=C=C=/≡C–C≡C– in-plane ring alternation mode. This choice for ω* ensures that both the slow (classical) and fast (quantum) modes are sampled equally accurately in the PIMC procedure (a full set of frequencies of the structures is given in the Supporting Information).

Subsequently, we define the best level of path integration by setting Δβω* = 0.1 and name this level QM5. Then, four progressively lower levels of treatment were investigated for identifying convergence of the key observables with *L* and simultaneously for measuring the importance of quantum effects in the vibrational dynamics, namely, QM4, QM3, QM2, and QM1, roughly corresponding to the quantum control factors Δβω* ≈ 0.2, 0.4, 0.8 and 1.6, that is gradually approaching the classical regime, and the actual integer values of *L* associated with them. At some temperatures, the values of Δβω* are slightly different for the same QM level due to the integer number of slices *L* (see Table 1 for exact values).

**Table 1:** Path Integration Parameters for the Five Quantum Regimes Considered in the Study: The Number of Path Slices *L*, the Number of Adjacent Slices *j* to be Moved Simultaneously, and the Quantum Control Factor Δβω* (See the Text), Where the Characteristic C=C Stretch Frequency ω* is 2025 cm^–1^ Corresponding to the Cumulene/Acetylene =C=C=C=/≡C–C≡C– Ring Alternation Mode^a^

*T*	QM1		QM2		QM3		QM4		QM5	
	*L* (*j*)	Δβω*	*L* (*j*)	Δβω*	*L* (*j*)	Δβω*	*L* (*j*)	Δβω*	*L* (*j*)	Δβω*
600	4 (1)	1.21	8 (2)	0.61	12 (4)	0.40	24 (8)	0.20	48 (16)	0.12
500	4 (1)	1.45	8 (2)	0.73	16 (4)	0.36	32 (8)	0.18	64 (16)	0.09
400	4 (1)	1.82	8 (2)	0.91	16 (4)	0.46	32 (8)	0.23	64 (16)	0.11
300	8 (1)	1.21	12 (2)	0.81	24 (4)	0.40	48 (8)	0.20	96 (16)	0.12
200	8 (1)	1.82	16 (2)	0.91	32 (4)	0.46	64 (8)	0.23	128 (16)	0.11
100	16 (1)	1.82	32 (2)	0.91	64 (4)	0.46	128 (8)	0.23	256 (16)	0.11

a The temperature *T* is given in Kelvin.

To avoid the inevitable slowdown of the MC process at large *L*,^34,35,41^ we make use of nonlocal path sampling techniques in the form of the staging algorithm defined by the number of path links *j* ≥ 1 that are displaced simultaneously with a randomly chosen pair of reference links.^42^ The exact length of a *j*-segment is adjusted to maintain the Metropolis acceptance ratio on the 40–60% range, although in some cases with very small *L*, lower acceptance ratios are allowed. These parameters are summarized in Tables 1 and 2.

**Table 2:** Ro-Vibrational Enthalpy Estimates *U* (in cm^–1^ Relative to the *D*_5*h*_ Global Minimum) and the Metropolis Acceptance Ratios, acc. (in %), Averaged over Four Independent 100,000 Monte Carlo Cycle Runs, with Each Following an Initial Equilibration with 5000 MC Cycles and Using the Symmetric Cyclic *D*_5*h*_–*D*_10*h*_–*D*_5*h*_′–*D*_10*h*_–*D*_5*h*_ Coordinate as the Initial Path (See the Text)^a^

*T*	QM1		QM2		QM3		QM4		QM5	
	*U*	acc.	*U*	acc.	*U*	acc.	*U*	acc.	*U*	acc.
600	14,239	40	14,859	53	14,958	46	15,012	52	15,008	55
500	12,750	30	13,540	44	13,776	53	13,853	58	13,891	61
400	11,215	19	12,206	32	12,629	41	12,690	46	12,714	50
300	10,907	42	11,320	39	11,619	48	11,712	53	11,760	56
200	9420	20	10,439	32	10,808	41	10,877	47	10,940	50
100	8895	20	9932	32	10,286	41	10,387	47	10,438	50

a The temperature *T* is given in Kelvin.

## 3 Results and Discussion

### 3.1 Properties of the Cyclo[10]carbon PES

Based on the preliminary tests described in Section 2.1, we confine our discussion of the PIMC calculations for temperatures up to 600 K as the higher temperature PIMC results may, first of all, not be reliable for **PES_B1**, and second, due to the presence of the triplet cyclo[10]carbon at 15,822 cm^–1^ above the global minimum, appear to suggest interference of the virtual triplet vibrational states at temperatures just below 1000 K. As an aside, our calculations and those of others^43^ place the triplet cyclo[10]carbon several kcal/mol below its linear triplet counterpart (see the Supporting Information). This is despite the fact that, to the best of our knowledge, experimental measurements are presently available only for the linear conformer in its triplet electronic state.^43,44^

To better estimate the lower bound for the temperature of the singlet–triplet contact, we performed a search for the minimum on the seam of crossing (MSX) of the S0/T1 surfaces. The search located a cyclic triplet *D*_2*h*_ structure with the energy 16,140 cm^–1^, or equivalently 968 K assuming equipartitioning of energy, above the global *D*_5*h*_ minimum. This calculation suggests that high-temperature simulations on the singlet PES should be coupled to the triplet PES to account for nonadiabatic effects. It further shows that the S0/T1 MSX is only 320 cm^–1^ above the T1 *D*_2*h*_ global minimum, well below the zero-point vibrational energy on the T1 PES. In other words, the cyclo[10]carbon in its triplet state is expected to be a metastable species due to the presence of an extremely low MSX with S0 while being mediated by spin–orbit-induced intersystem crossing, examined computationally by others for carbon clusters,^45^ to the much more stable S0 ground state (see the Supporting Information for S0 and T1 PES profiles).

The possible path of decay of the singlet cyclo[10]carbon to its linear configuration basin has not been explicitly considered here since the linear configuration is known to be much higher in energy, and our own calculations, summarized in the Supporting Information, agree with the previous computational studies,^43^ placing the linear isomer ∼2.4 eV above the *D*_5*h*_ global minimum. The ring-to-linear transition state (RL-TS) was also identified to be very high energetically, ∼5 eV, and in a nonplanar configuration, which suggests involvement of out-of-plane vibrational modes in transition to linearity, a feature that will be examined in more detail in Section 3.3.

**Figure 2:**
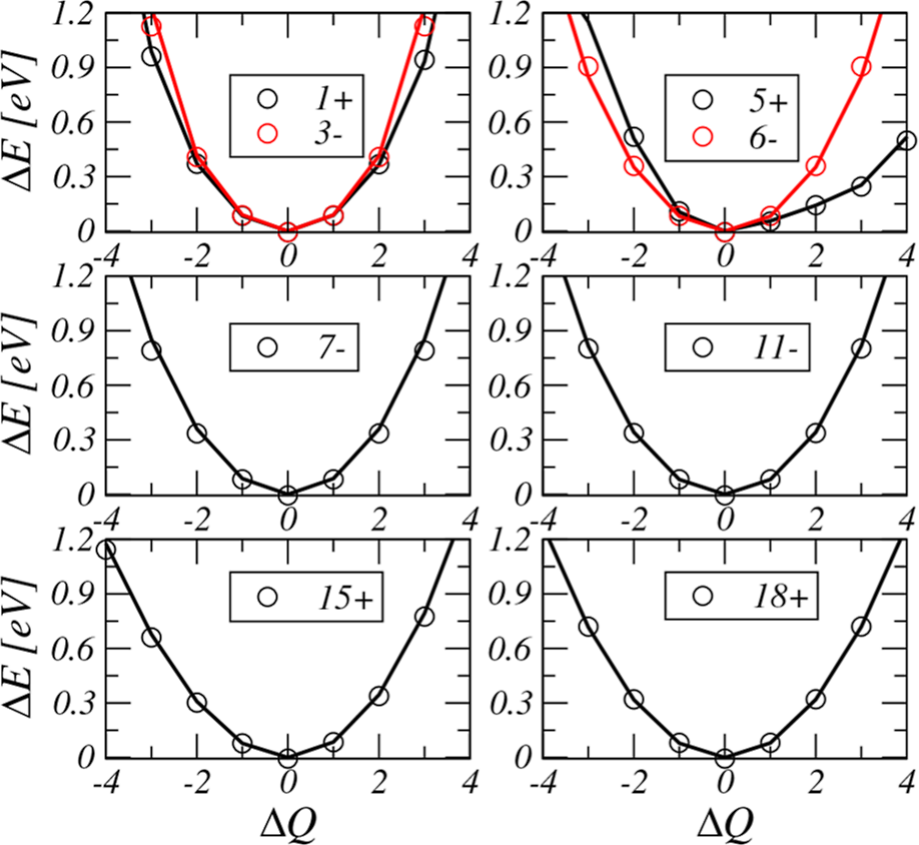
One-dimensional cuts of the potential energy calculated with **PES_B1** (solid lines) and the corresponding *ab initio* data (open circles) along a few representative normal modes with displacement Δ*Q* in the increasing frequency order, as indicated by the corresponding mode number with the in-plane (+) and out-of-plane (−) reflection parity index. Δ*Q* = 0 corresponds to the *D*_5*h*_ configuration (additional cuts and definitions are given in the Supporting Information). The *ab initio* data are the τHCTH/cc-pVQZ energies adjusted to reproduce the benchmark *D*_5*h*_–*D*_10*h*_ inversion barrier (see the text for details).

Initially, **PES_B1** was trained to reproduce the exact *D*_5*h*_–*D*_10*h*_ isomerization barrier of 812 cm^–1^.^27^ The fit recovered the barrier to within ∼56 cm^–1^ yielding a value of 756 cm^–1^, and the isomerization path is reproduced by **PES_B1** very closely on the energy span of 0–5000 cm^–1^, as can be seen in Figure 1, with the cumulative RMSE of 29 cm^–1^. Additionally, cuts along all the normal mode coordinates were examined, and some of these are summarized in Figure 2, whereas the full set is shown in the Supporting Information. One can observe a very good performance of **PES_B1** for energies up to 1.2 eV in each normal coordinate, especially considering that these data points were not part of the training set.

Before moving on to the PIMC simulations, we examined the HOMO–LUMO gap, a property that measures excited state mixing and JTE strength, along two normal modes that closely correlate with inversion coordinate *X* (mode-5) and out-of-plane distortion (mode-11). For the inversion coordinate, the calculations show the HOMO–LUMO gap to be 3.9 eV at the *D*_5*h*_ global minimum and smoothly decreasing to 3.5 eV at the *D*_10*h*_ inversion transition state, consistent with a second-order JTE resulting from a very weak mixing of an excited electronic state at the high-symmetry *D*_10*h*_ structure. Scanning this mode in the direction away from *D*_10*h*_, the HOMO–LUMO gap closes much more rapidly becoming 1.7 eV at vibrational energies 4–5 eV above the global minimum, suggesting fairly strong mixing of excited states at high temperatures. Similar behavior was recorded for the out-of-plane coordinate (mode-11) where the HOMO–LUMO gap shrinks from 3.9 eV at the planar equilibrium to about 2 eV at vibrational energies approaching 2–3 eV regime as the ring is distorted out of plane. From these, we can infer a correlation between the increasing temperature and the closing of the HOMO–LUMO gap, indicative of excited state mixing.

### 3.2 PIMC Simulations

Our approach to path integration is to generate an initial path symmetrically so as to connect the four points *D*_5*h*_–*D*_10*h*_–*D*_5*h*_′–*D*_10*h*_ in a circulant manner, slicing each quarter-path in *L*/4 fragments and interpolating between them using the linear rule. We note that *D*_5*h*_ and *D*_5*h*_′ are two identical global minima related by a pseudorotation, which is a true rotation by 72°, or equivalently by a one-index cyclic permutation of the nuclear indices, a property contained in the permutation-invariant **PES_B1** used in the calculations. This choice for the initial path allows us to examine the final Monte Carlo distributions in relation to an exactly symmetric *D*_5*h*_–*D*_5*h*_′ double-well density. In the definition of the path described above, the minimal value of *L* is 4. Such a partitioning of the initial path ensures symmetrical sampling of the phase space in the early stages of the simulation, and it leads to an unbiased distribution after an extended initial Metropolis Monte Carlo run. For each temperature we run four independent samples, at first equilibrating each sample using 5000 steps, and then generating ensembles of length 100,000 steps and averaging the results. Table 2 summarizes the outcomes of these simulations for each QM level and the corresponding temperature.

**Figure 3:**
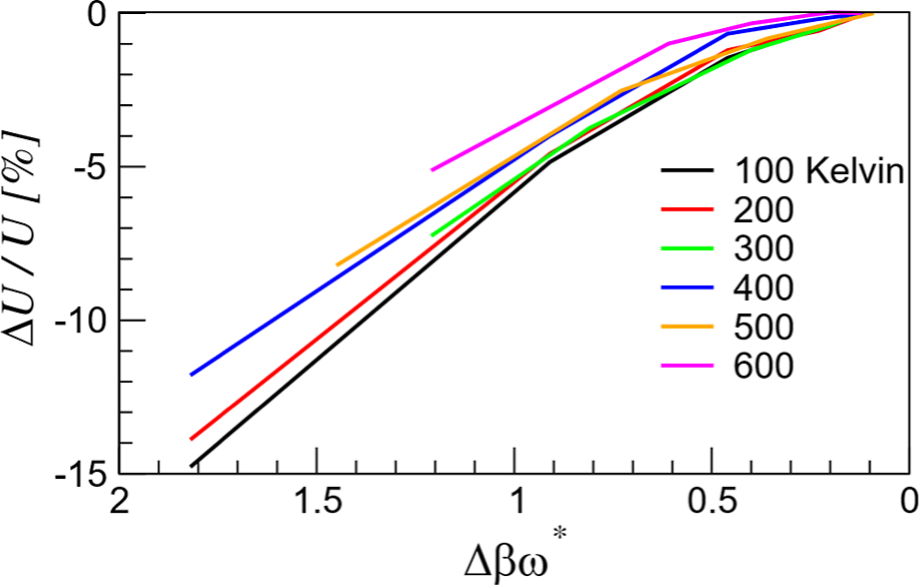
Test of path integration convergence using the ro-vibrational enthalpy as a function of the quantum control factor: the product of the reduced inverse temperature Δβ ≡ β/*L* and the characteristic frequency ω* = 2025 cm^–1^. The quantity on the vertical axis is the percent ratio of the enthalpy difference Δ*U* = *U*(Δβω*) – *U*(0.1) (between the given level of path integration and the highest level of path integration for which Δβω* ≈ 0.1) to the enthalpy at the highest level of path integration.

A useful test to examine convergence with respect to path integration parameter *L* is the enthalpy measurement against quantum control factor Δβω*, whose exact values for each temperature are reported in Table 1. Figure 3 illustrates the enthalpy convergence rates for the six temperatures. For these test cases, a convergence is clearly visible, leading to the QM5 level, where Δβω* = 0.1. This result supports our initial assumption that Δβω* ≈ 0.1 is a sufficiently accurate level of path integration to describe the quantum effects in C_10_. As expected, the importance of quantum effects is more significant at the lowest temperatures, e.g., 100 and 200 K, where the enthalpy calculated using the QM1 level of path integration deviates from that of QM5 by ∼15%. For the higher temperatures (*T* > 300 K), the deviation between QM1 and QM5 is, on average, between 5 and 12% depending on the actual value of Δβω* (note the slight differences in the actual values of Δβω* for the same QM level at different temperatures).

To finalize this analysis, we summarize the data for the enthalpy *U* and the specific heat *C*_V_ ≡ ∂*U*/∂*T* for the QM5 level as functions of the temperature (both shown in Supporting Information along with corresponding data for the rigid rotor harmonic oscillator). We note in passing that the enthalpy at 600 K reaches the energy region that has a relatively low density of training data points. This partially explains our preliminary observations that some of the higher temperature simulations, excluded from the present analysis, e.g., 800 K and higher, occasionally resulted in sampling of unphysical configurations.

**Figure 4:**
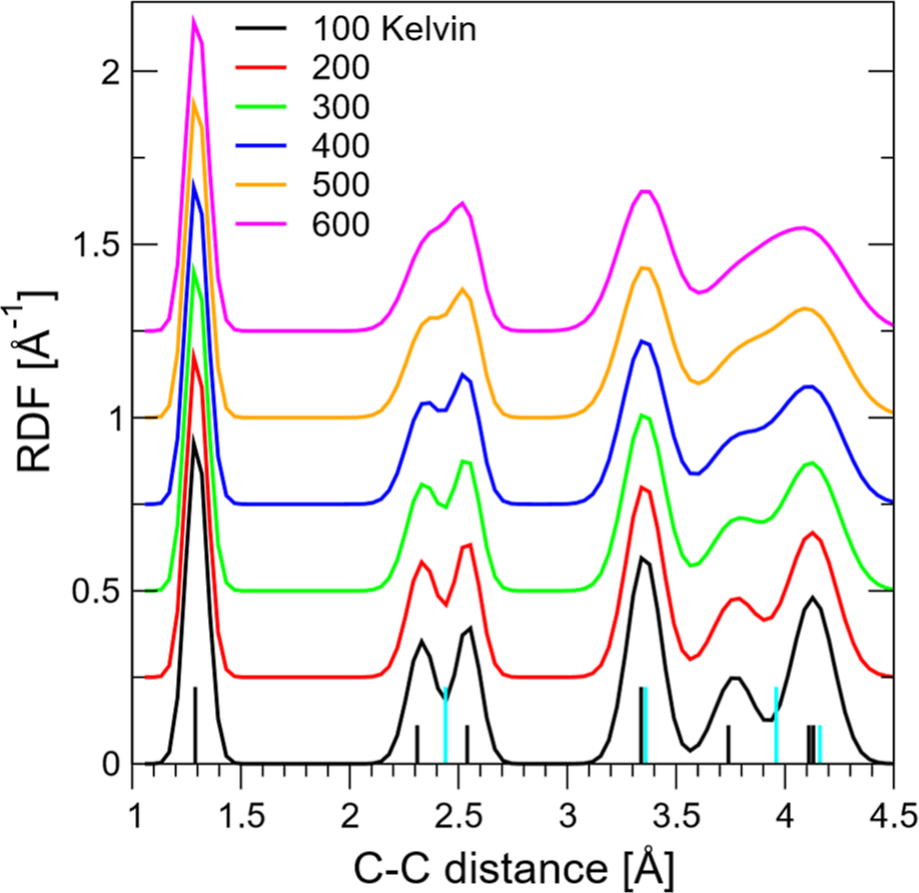
Normalized RDF as colored curves calculated at the highest level of path integration QM5 for the six temperatures; the black/cyan vertical sticks are the internuclear distances taken at the *D*_5*h*_/*D*_10*h*_ stationary points, respectively. The characteristic structural regions are **R0** on [1, 1.5] Å which is the C=C double bond distance; **R1** on [2.1, 2.7] Å, which corresponds to the second-near-neighbor C–c–C pairs which describe the *D*_5*h*_ configuration; and **R2** on [3, 4.5] Å which involves all other pair distances. As the temperature increases, the distinction between *D*_5*h*_ and *D*_10*h*_ manifested as the double peak in **R1** becomes progressively less clear.

Turning to the main objective of examining the JTE at nonzero temperatures, we discuss the RDFs calculated at the six temperatures. For this analysis, we use the results obtained with the most accurate level of path integration: QM5. These calculations are presented in Figure 4 where we identify the three main regions of the C–C distance: the C=C double bond region **R0** on [1, 1.5] Å, the next-near-neighbor C–c–C region **R1** on [2.1, 2.7] Å, and region **R2** describing all other pairs broadly spread over [3, 4.5] Å. We first point out the obvious feature of **R0** which identifies the cyclo[10]carbon as a cumulene, with the thermally averaged C=C bond being ∼1.3 Å virtually independent of temperature. There is no evidence of the acetylenic signature, even at the high temperatures, in contrast to recently reported DFT calculations with quantum vibrational corrections applied at 0 K, suggesting the existence of a low-energy *D*_5*h*_ acetylenic structure.^17^ The key signature pointing to the *D*_10*h*_ → *D*_5*h*_ symmetry-breaking, as seen most distinctly in the 100 K RDF, shows up in the **R1** region where the double-headed feature clearly identifies the two different C–C groups peaked at 2.35 Å (**R1.1**) and 2.55 (**R1.2**) Å, respectively, as illustrated in Scheme S1 by two overlapping pentagons. The side of the larger pentagon, 2.55 Å, is very close to the sum of two C=C bonds measured on **R0** at 1.3 Å inferring a nearly straight C–C–C angle, a feature of the pentagonal geometry in cyclo[10]carbon. Accompanying this basic structure is the finer structure in region **R2**, where three groups of distances are identified at 3.35 (**R2.1**), 3.75 (**R2.2**), and 4.15 (**R2.3**) Å. They correspond to the C–c–c–C, C–c–c–c–C, and C–c–c–c–c–C pairs. The 100 K RDF closely reflects our previous DMC calculations at 0 K.^28^ It shows that the pentagonal *D*_5*h*_ structure is very stable at 100 K. Continuing to observe the evolution of the RDF as the temperature increases, we may conclude that the *D*_5*h*_ structure persists up to 200 K. It then gradually wanes and, commencing at 300 K, approaches that of a loose *D*_10*h*_ ring at *T* > 400 K.

**Figure 5:**
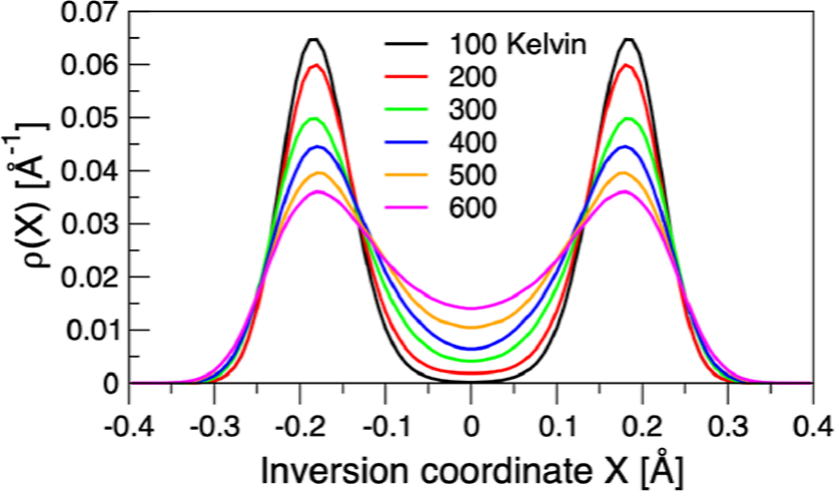
Projection of the full dimensional density ρ{**x**} calculated at the QM5 level onto the double-well inversion coordinate *X*, for each of the six temperatures. In the vicinity of either of the two *D*_5*h*_ minima, *X* = ±0.2 Å; at a *D*_10*h*_ configuration *X* = 0. A deep tunneling regime, with the probability of the *D*_10*h*_ structure being less than 10% is identified at 100 and 200 K (see also Table 3), gradually waning as the temperature increases.

The broadness of the **R1** and **R2** regions in the RDF suggests a fair amount of *D*_5*h*_ symmetry at all of the temperatures. To scrutinize the results closer, we take the approach used in our earlier work and project the multidimensional density ρ{**x**} onto the inversion coordinate *X* defined by the difference of an innermost radius (the small pentagon in Scheme S1) and an outermost radius (the big pentagon in Scheme S1) relative to the centroid and averaged over the ring. We label this projected density by ρ(*X*). At either of the two *D*_5*h*_ minima, we have |*X*| = ∼0.2 Å; at a *D*_10*h*_ configuration, we have *X* = 0. Figure 5 shows the projected PIMC densities for all of the temperatures. The obvious double-well structure of the density is due to the JTE and is most prominent at the lower temperatures (<200 K), which we may characterize as a deep tunneling regime. Starting at 300 K, the JTE clearly weakens, as reflected in the flattening of the ρ(*X*) curve and the increasing probability at *X* = 0. For a quantitative analysis, we define a *D*_10*h*_ structure as one confined to the [−δ, δ] segment on *X* for some appropriate value of δ, and integrate ρ(*X*) inside and outside the region. This approach gives us an estimate of the relative probability of finding a cyclo[10]carbon in a *D*_5*h*_ or *D*_10*h*_ structure. For instance, choosing δ = 0.1 Å leads to the maximal deviation of ±10° from the characteristic *D*_10*h*_ angle of 144° which we interpret as a reasonably accurate partition between the *D*_5*h*_ and *D*_10*h*_ symmetry basins. The calculations of the relative probabilities using the above definitions and parameters are summarized in Table 3. These results show the *D*_5*h*_ component decreasing steadily with increasing temperature, which correlates with the JTE decreasing at about the same rate. For a more clear perspective of the ring temperature evolution, we examine the rate of growth of the *D*_10*h*_ contribution as summarized in Table 3. Notably, at 600 K, the *D*_10*h*_ character reaches ∼26%, a fraction being fairly close to its expected value of ∼33% assuming a top hat density curve in the high-temperature limit (illustrated in the Supporting Information), i.e., significantly above the *D*_10*h*_ barrier, and the equipartitioning of the density along *X* on the regions [−0.3, δ], [−δ, δ], and [δ, 0.3] with δ = 0.1 Å. In other words, the ring is expected to be a nearly symmetric decagon at temperatures above 600 K. This trend may be further illustrated by a composite picture of overlaid configurations recorded along the PIMC “trajectories”, as shown separately in the Supporting Information. Convergence of the *D*_5*h*_ structure to the *D*_10*h*_ structure is more evidently captured by a visual inspection showing that already at 300 K the decagonal *D*_10*h*_ shape has emerged.

**Table 3:** Decomposition of the Projected Density ρ(*X*) Calculated at the QM5 Level of Path Integration Using the (*D*_5*h*_/*D*_10*h*_) Partition of the Inversion Coordinate *X* with δ = 0.1; See the Text for Details^a^

*T*/Kelvin	% *D*_5*h*_	% *D*_10*h*_	*D*_10*h*_ ratio
100	96.4	3.6	1.0
200	93.0	7.0	1.9
300	87.7	12.3	3.4
400	83.2	16.8	4.7
500	78.2	21.8	6.1
600	74.2	25.8	7.2

a The ratio of the *D*_10*h*_ density to its value at 100 K is also shown.

### 3.3 Deviation from Planarity

In the above analysis, we have tacitly assumed a planar C_10_ ring, that is, without explicitly measuring the extent of the out-of-plane motions. We have stated the nonplanarity of the RL-TS and briefly speculated about the out-of-plane vibrational modes interfering with the in-plane normal modes, e.g., by lifting the inversion, reflection, and rotation symmetries in the *D*_5*h*_ and *D*_10*h*_ point groups. To examine the degree of deviation of the ring from planarity, particularly as a function of the temperature, we measure the average distance of the nuclei to a plane fitted in a least-squares sense through the 10 carbon positions at each configuration along the Monte Carlo sequence. The details of this procedure are given in the Supporting Information.

**Figure 6:**
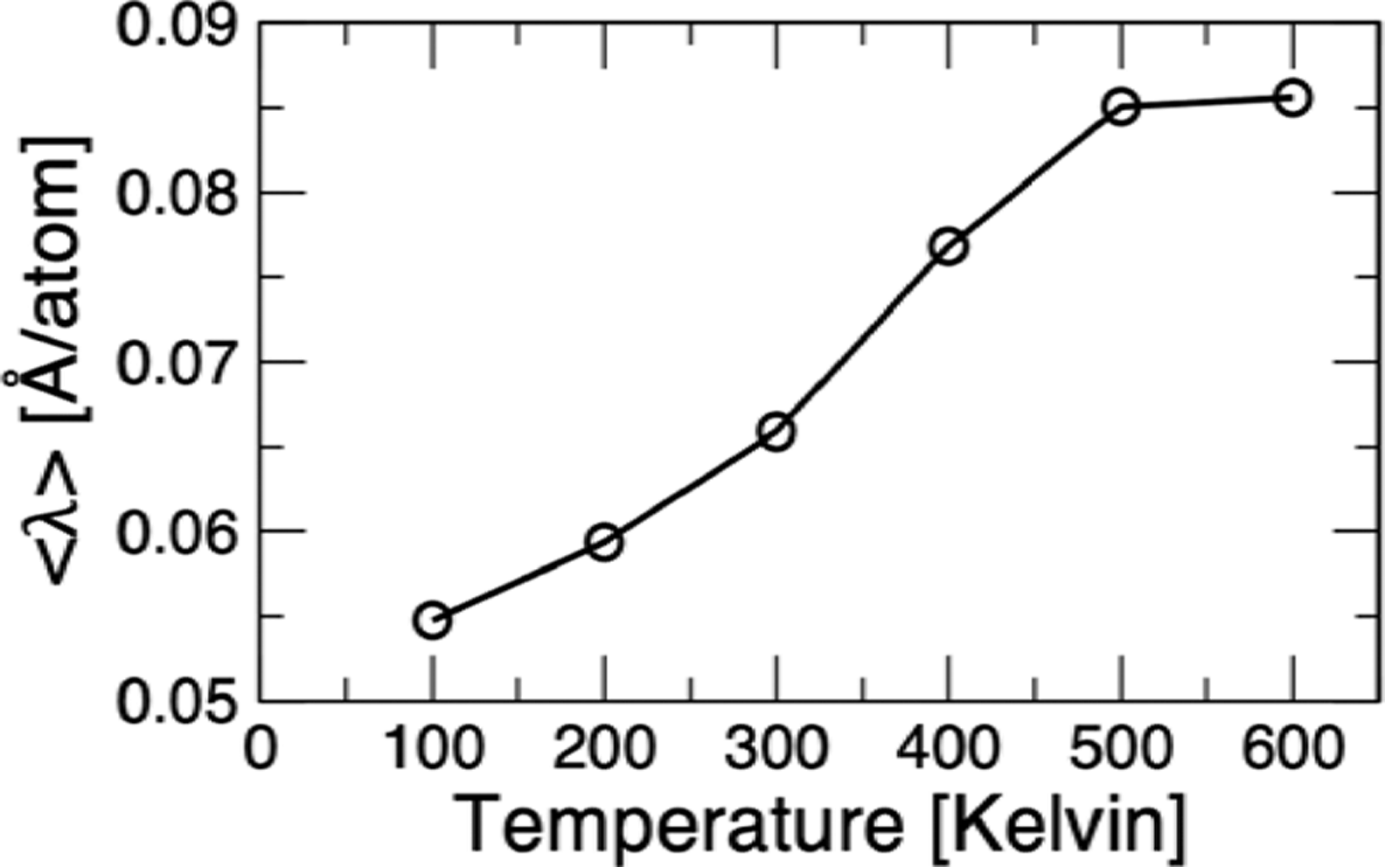
Average deviation of the C_10_ ring from planarity ⟨λ⟩ measured in units of Å/atom plotted as a function of temperature and calculated using PIMC with the QM5 level of integration. The definition of ⟨λ⟩ and other underlying quantities are given in the Supporting Information.

In Figure 6, we summarize the average deviation from planarity, λ, as a function of temperature. As seen, the deviation increases monotonically but has a curvature which shows a moderate rise, an inflection point, at ∼200 K, followed by a much sharper gain on [300, 500] Kelvin and culminating with an evident lop-off on reaching 600 K. While these features are challenging to interpret, the absolute value of the deviation can be quantified as being fairly small; that is, at 600 K it is ∼0.085 Å/atom. The calculated average deviation from planarity is appreciably smaller than the deformation of the ring along the 1-D *X*-coordinate from the *D*_10*h*_ to the global minimum *D*_5*h*_ configuration, a value of ∼0.2 Å. This observation also sheds some light on the nature of the RL-TS structure, which is nonplanar and has a very high energy, presently calculated to be ∼5 eV, above the global minimum. Its deviation from planarity λ_RL-TS_ = 0.11 Å/atom is slightly greater than that of the thermally averaged counterparts at the temperatures simulated in the present study. We tentatively conclude that the thermally induced out-of-plane distortions in cyclo[10]carbon incur a substantially higher energy penalty than those involving the in-plane motions.

## 4 Conclusions

In the work reported here, we have described extensive path integral Monte Carlo simulations of the singlet cyclo[10]carbon at temperatures ranging from 100 to 600 K using a permutationally invariant PES, labeled as **PES_B1**. The PES was fitted to τHCTH/cc-pVQZ data and modified to reproduce the *D*_5*h*_–*D*_10*h*_ isomerization barrier of 812 cm^–1^ benchmarked previously^27^ at the all-electron CCSDT(Q)/CBS level of theory. The simulations show that the cyclo[10]carbon distorted by the JTE from the regular decagon to a pentagonal configuration of *D*_5*h*_ symmetry is indeed the preferred vibrationally averaged configuration not only at 0 K (as demonstrated in our earlier publication)^28^ but also at the temperatures up to ∼300 K at which point a loose *D*_10*h*_ structure emerges and becomes progressively more viable as the temperature increases further. Our results partially validate the pioneering LDA-DFT molecular dynamics simulations^29^ (a lower level of theory than the present) which nevertheless identified a stable *D*_5*h*_ structure at 77 K but a floppy *D*_10*h*_ structure at 200 K. Specific points addressed in the present PIMC simulations can be summarized as

(i) Inclusion of quantum vibrational effects beyond the normal mode treatment of the *D*_5*h*_–*D*_10*h*_ reaction coordinate (Figure 1A) is key to recovering the experimentally measured structure of singlet cyclo[10]carbon;
(ii) Insignificant tunneling through the *D*_10*h*_ barrier, i.e., less than 10% probability as defined in the present study, is observed at temperatures below 200 K;
(iii) The rate of barrier penetration continues to increase from 200 K and up reaching 26% at 600 K;
(iv) The analysis of the deviation from planarity reveals the ring’s structure to be close to planar with the maximum average deviation found to be ∼0.09 Å/atom at 600 K, somewhat smaller than the 0.11 Å/atom deviation of the high-energy (∼5 eV above the global minimum) ring-to-linear transition state. The apparent stiffness of the out-of-plane deformational vibrations at the temperatures up to 600 K is presently left open for interpretation and may be understood via an energy decomposition analysis;
(v) As a final remark, we speculate on the (non)existence of the triplet state cyclo[10]carbon. Our calculations reveal a low-energy S0/T1 point of crossing, which is well below the T1 zero-point vibrational energy level. Therefore, it is expected that the cyclo[10]carbon triplet, when isolated under experimental conditions, will be metastable. Molecular dynamics simulations, whether quantum or classical, on S0 and T1 surfaces while mediated by spin–orbit coupling can be instrumental in addressing this issue.

## Supporting Information Available

Structure, energetics, and vibrational frequencies; 1-D cuts of the potential energy; thermodynamic functions; visual analysis of the ring evolution with temperature; and calculations of deviation from planarity (PDF)
