# Supplementary material for: First-Principles Path Integral Monte Carlo Studies of the Pseudo Jahn–Teller Effect in the Aromatic Cyclo[10]carbon
Source: J Phys Chem A. 2025 Feb 24;129(9):2238–46. doi: 10.1021/acs.jpca.4c08620 (PMC11891903; doi:10.1021/acs.jpca.4c08620)
Supplement: Supplementary file 1 — jp4c08620_si_001.pdf [file jp4c08620_si_001.pdf]

Supporting Information for

First Principles Path Integral Monte Carlo Studies of the Pseudo Jahn-Teller Effect in the Aromatic Cyclo[10]carbon

Anna H. James,[1] Martina Kaledin,[1,*] Alexey L. Kaledin[2,*]

[1] *Department of Chemistry & Biochemistry, Kennesaw State University, 370 Paulding Ave NW, Box # 1203, Kennesaw, Georgia, 30144 USA*

[2] *Cherry L. Emerson Center for Scientific Computation and Department of Chemistry, Emory University, 1515 Dickey Drive, Atlanta, Georgia, 30322 USA*

Table of contents

S-1. Structure, energetics and vibrational frequencies of C_{10} S2

S-2. One-dimensional cuts of the potential energy calculated with PES_B1......................S9

S-3. Summary of thermodynamic functions S10

S-4. Visual analysis of the ring evolution with temperature S12

S-5. Calculation of the average deviation from planarity S13

S-6. DVR calculations in 1-D S14

S-7. References S15

S-1. Structure, energetics and vibrational frequencies of C_{10}

All electronic structure calculations were carried out with Gaussian 09.[1] The search for the MSX was performed with the MECP code.[2]

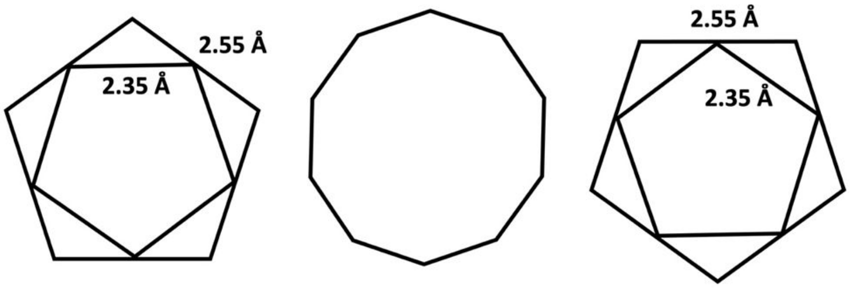

Scheme S1. A visual representation of the D_{5h} ⇔ D_{10h} ⇔ D_{5h}' reaction using two overlapping pentagons constructed with the average values of the C-c-C distances calculated at 100 K and taken from Figure 4 of the main text. The D_{10h} transition state structure is a regular decagon placed in the middle. The vertices of the two pentagons represent the nuclei of C_{10} cyclically permuted by one nuclear index. The numbers of the unique internuclear distances in each of the regions are: **R0** = 10; **R1** = 10; and for three subregions within **R2** (refer to Figure 4): **R2.1** = 10, **R2.2** = 5, **R2.3** = 10, in the order of increasing distance.

Table S1. Energetics of the C_{10} carbon cluster at the τHCTH/cc-pVQZ level of theory with E and E_0 as total electronic and ZPVE corrected energies.

State	Symmetry	Shape	Nimag	E (Hartree)	ΔE (cm^{-1})	E_0 (Hartree)	ΔE_0 (cm^{-1})
S0	D_{5h}	ring	0	-380.676568	0	-380.629442	0
S0	D_{10h}	ring	1	-380.673185	742	-380.627562	413
S0	$D_{\infty h}$	linear	0	-380.588267	19380	-380.545996	18314
S0	C_1	horseshoe	1	-380.493830	40106	-380.452728	38784
T1	D_{2h}(1)	ring	0	-380.604477	15822	-380.560669	15094
T1	$D_{\infty h}$	linear	0	-380.594300	18056	-380.552114	16972
T1	D_{2h}(2)	ring	1	-380.591232	18729	-380.551838	17032
T1	C_1	horseshoe	1	-380.502523	38198	-380.461902	36771
MSX	D_{2h}	ring	N/A	-380.603027	16140		

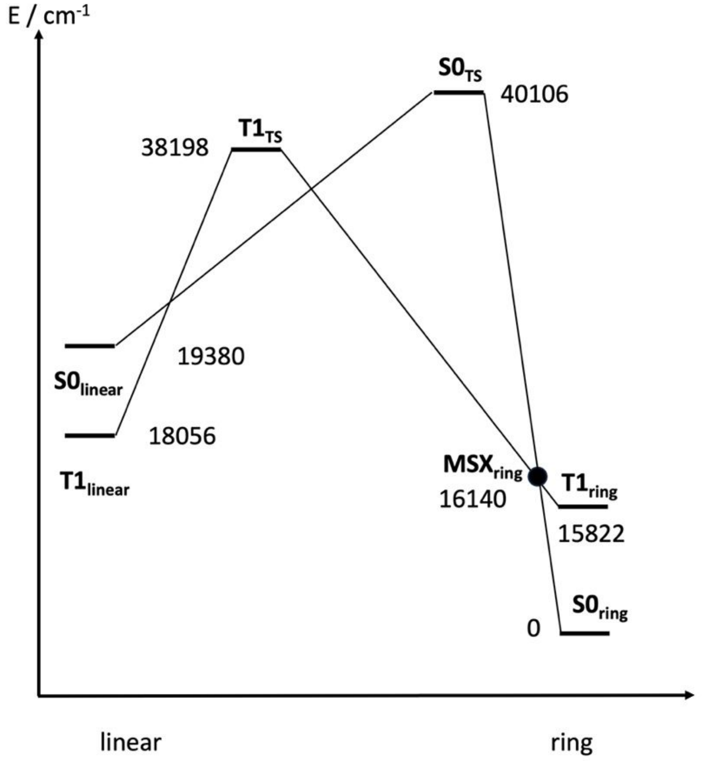

Figure S1. A schematic plot of the key structures of C_{10}, calculated with τHCTH/cc-pVQZ, along the ring-to-linear reaction path, represented by the horizontal axis. The vertical axis is the electronic energy in cm^{-1}. In the "ring" basin, there are the S0 and T1 global minima. The "linear" basin contains the corresponding linear minimum. The lowest energy point on the seam of the S0/T1 crossing is labeled MSX and has a D_{2h} ring structure. The two ring-opening transition states, $S0_{TS}$ and $T1_{TS}$, respecively, resemble slightly twisted horseshoe structures.

Table S2. XYZ coordinates (Å) of the D_{5h} global S0 minimum at the τHCTH/cc-pVQZ level of theory.

C	0.000000	1.968490	0.000000
C	-1.271553	1.750124	0.000000
C	-1.872631	0.608440	0.000000
C	1.271600	1.750097	0.000000
C	-2.057524	-0.668456	0.000000
C	1.872631	0.608442	0.000000
C	2.057477	-0.668501	0.000000
C	-1.157323	-1.592768	0.000000
C	-0.000027	-2.163057	0.000000
C	1.157349	-1.592811	0.000000

Table S3. XYZ coordinates (Å) of the D_{10h} inversion S0 TS at the τHCTH/cc-pVQZ level of theory.

C	0.000000	0.000000	2.079524
C	0.000000	1.222410	1.682440
C	0.000000	1.977964	0.642660
C	-0.000000	-1.222410	1.682440
C	0.000000	1.977964	-0.642660
C	-0.000000	-1.977964	0.642660
C	-0.000000	-1.977964	-0.642660
C	0.000000	1.222410	-1.682440
C	0.000000	0.000000	-2.079524
C	-0.000000	-1.222410	-1.682440

Table S4. XYZ coordinates (Å) of the $D_{\infty h}$ (linear) S0 minimum at the τHCTH/cc-pVQZ level of theory.

C	0.000000	0.000000	-5.776566
C	0.000000	0.000000	-4.480902
C	0.000000	0.000000	-3.189559
C	0.000000	0.000000	-1.917719
C	0.000000	0.000000	-0.636681
C	0.000000	0.000000	0.636683
C	0.000000	0.000000	1.917716
C	0.000000	0.000000	3.189563
C	0.000000	0.000000	4.480897
C	0.000000	0.000000	5.776568

Table S5. XYZ coordinates of ring-to-linear S0 TS at the τHCTH/cc-pVQZ level of theory.

C	-1.758119	-1.218348	-0.037955
C	-0.763893	-2.015815	0.070299
C	0.523005	-1.890337	0.035304
C	-2.316820	-0.050907	-0.026026
C	1.594029	-1.199797	0.012563
C	-1.813738	1.122165	-0.083842
C	-0.984072	2.132857	-0.050631
C	2.349392	-0.153288	-0.029992
C	2.898867	1.030417	-0.103767
C	0.271349	2.243054	0.214047

Table S6. XYZ coordinates (Å) of the D_{2h} (ring) T1 minimum at the τHCTH/cc-pVQZ level of theory.

C	1.714618	1.051531	0.000060
C	2.521294	-0.000347	0.000095
C	1.714440	-1.052198	-0.000019
C	0.646898	1.757385	-0.000736
C	0.646017	-1.757009	0.000676
C	-0.646889	1.759033	0.001143
C	-1.714141	1.052437	-0.001127
C	-0.647685	-1.759352	-0.000931
C	-1.714442	-1.051791	0.000061
C	-2.520110	0.000310	0.000778

Table S7. XYZ coordinates (Å) of the $D_{\infty h}$ (linear) T1 minimum at the τHCTH/cc-pVQZ level of theory.

C	0.000000	0.000000	-5.775416
C	0.000000	0.000000	-4.477963
C	0.000000	0.000000	-3.188366
C	0.000000	0.000000	-1.916250
C	0.000000	0.000000	-0.636702
C	0.000000	0.000000	0.636703
C	0.000000	0.000000	1.916249
C	0.000000	0.000000	3.188369
C	0.000000	0.000000	4.477958
C	0.000000	0.000000	5.775419

Table S8. XYZ coordinates of the D_{2h} T1 TS at the τHCTH/cc-pVQZ level of theory.

C	0.000000	0.000000	1.906625
C	0.000000	1.234413	1.532200
C	0.000000	2.195963	0.687405
C	0.000000	2.195963	-0.687405
C	0.000000	1.234413	-1.532200
C	0.000000	0.000000	-1.906625
C	0.000000	-1.234413	-1.532200
C	0.000000	-2.195963	-0.687405
C	0.000000	-2.195963	0.687405
C	0.000000	-1.234413	1.532200

Table S9. XYZ coordinates of ring-to-linear T1 TS at the τHCTH/cc-pVQZ level of theory.

C	2.110203	0.081733	0.025485
C	1.949400	-1.189872	-0.030844
C	0.837217	-1.862127	-0.073327
C	1.754414	1.321290	0.078297
C	-0.430783	-1.951856	-0.066636
C	0.593586	1.868746	0.069878
C	-0.674132	2.127606	0.039118
C	-1.659801	-1.532752	-0.033744
C	-2.590502	-0.680592	0.263937
C	-1.889603	1.817823	-0.272164

Table S10. XYZ coordinates of the S0/T1 crossing point, MSX at the τHCTH/cc-pVQZ level of theory.

C	0.000000	-1.696003	0.643757
C	0.000000	-1.018227	1.730580
C	0.000000	0.000000	2.588587
C	0.000000	-1.696581	-0.647912
C	0.000000	1.018227	1.730580
C	0.000000	-1.018341	-1.734478
C	0.000000	0.000000	-2.592219
C	0.000000	1.696003	0.643757
C	0.000000	1.696581	-0.647912
C	0.000000	1.018341	-1.734478

Table S11. Harmonic vibrational frequencies ω_n in cm^{-1} of the S0 structures at the τHCTH/cc-pVQZ level of theory.

	Global minimum	Isomerization TS	Linear geometry	Ring-opening TS
n	D_{5h}	D_{10h}	$D_{\infty h}$	C_1
1	186	313*i*	41	190*i*
2	187	197	41	65
3	231	197	99	112
4	231	233	99	148
5	379	233	168	170
6	397	303	168	215
7	428	411	242	257
8	428	412	242	278
9	464	419	354	332
10	465	419	354	344
11	469	421	408	401
12	469	421	459	406
13	515	451	459	435
14	515	452	502	496
15	819	799	502	524
16	1051	1056	511	780
17	1051	1057	511	957
18	1480	1520	786	1214
19	1480	1528	1137	1474
20	1575	1529	1462	1708
21	1912	1919	1763	1835
22	1912	1920	1950	1874
23	2025	2052	2028	1971
24	2025	2052	2110	2033
25			2147	

Table S12. Harmonic vibrational frequencies ω_n in cm^{-1} of the T1 structures at the τHCTH/cc-pVQZ level of theory.

	Global minimum	Linear geometry	Ring-opening TS	Isomerization TS
n	$D_{2h}(1)$	$D_{\infty h}$	C_1	$D_{2h}(2)$
1	57	41	216i	266i
2	166	41	84	180
3	183	101	131	189
4	192	101	151	192
5	232	170	166	194
6	317	170	214	262
7	372	243	279	300
8	395	243	296	361
9	397	355	352	381
10	405	355	355	412
11	433	409	372	427
12	434	457	406	446
13	437	457	453	446
14	479	500	471	484
15	807	500	489	603
16	899	510	806	765
17	1080	510	920	837
18	1240	787	1203	950
19	1519	1137	1445	1337
20	1571	1460	1650	1514
21	1774	1756	1795	1587
22	1849	1943	1823	1675
23	1901	2019	1974	1829
24	2086	2110	1985	1924
25		2146		

Table S13. Excited singlet states of C_{10} are calculated at the geometry of the D_{10h} transition state using the time-dependent τHCTH/cc-pVQZ level of theory. The symmetry labels correspond to the D_{2h} point group.

state	symmetry	Energy / eV	Energy / cm^{-1}
S1	B_{2u}	3.5568	28687
S2	B_{1u}	3.6705	29604
S3	B_{2g}	3.7408	30171
S4	B_{1g}	3.7480	30229

S-2. The one-dimensional cuts of the potential energy calculated with PES_B1

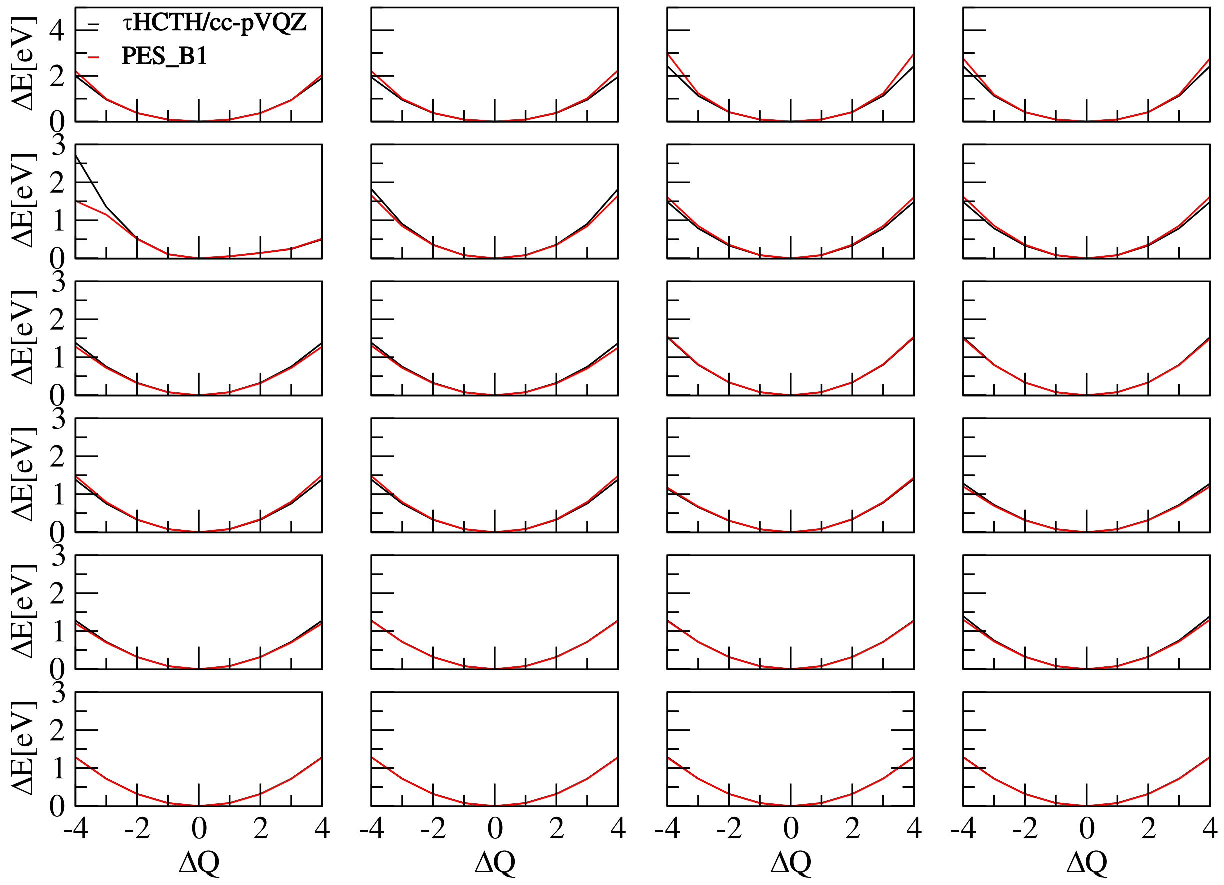

Figure S2. The 24 one-dimensional cuts of the potential energy of cyclo[10]carbon calculated with **PES_B1** (red curves) and the corresponding *ab initio* data (black curves) along the normal modes with coordinates ΔQ in the increasing frequency ω order from left to right starting at the upper left frame. The unit of ΔQ is $\sqrt{2E}/5\omega$ with E = 2 eV. ΔQ = 0 corresponds to the D_{5h} global minimum. The harmonic vibrational frequencies are provided in Table S11.

S-3. Summary of thermodynamic functions

The RDF is defined by directly using Eq. 7 and Gaussian binning with the pair distance r

$$\mathrm{RDF}(r) = \left\langle \frac{1}{L} \sum_{l=1}^{L} \sum_{i<j} exp\left(-\frac{1}{2} \left(\frac{r_{ij}^{(l)} - r}{\Delta r} \right)^2 \right) \right\rangle \qquad (S1)$$

where first sum runs over the L path slices and the second sum runs over all nucleus-pairs i and j with distance $r_{ij}^{(l)}$ apart.

The ro-vibrational enthalpy is derived using the kinetic energy estimator,[3]

$$U = \left\langle \frac{3N-3}{2\Delta\beta} - \frac{1}{2L} \sum_{l=1}^{L} \frac{|\mathbf{x}_l - \mathbf{x}_{l+1}|^2}{\Delta\beta^2} + \frac{1}{2L} \sum_{l=1}^{L} V(\mathbf{x}_l) \right\rangle \qquad (S2)$$

with the brackets in both of the above equations implying the Monte Carlo average. Specific heat is calculated by central differences using the data points T_i and their values $U(T_i)$,

$$C_V(T_i) = \frac{U(T_{i+1}) - U(T_{i-1})}{T_{i+1} - T_{i-1}} \qquad (S3)$$

The corresponding harmonic oscillator equivalents are

$$U^{\mathrm{H.O.}} = kT\left(\frac{\omega}{2kT}\right) \coth\left(\frac{\omega}{2kT}\right) \qquad (S4)$$

$$C_V^{\mathrm{H.O.}} = k\left(\frac{\omega}{2kT}\right)^2 \sinh^{-2}\left(\frac{\omega}{2kT}\right) \qquad (S5)$$

where we use $k = 0.69503877\mathrm{cm}^{-1}/\mathrm{Kelvin}$.

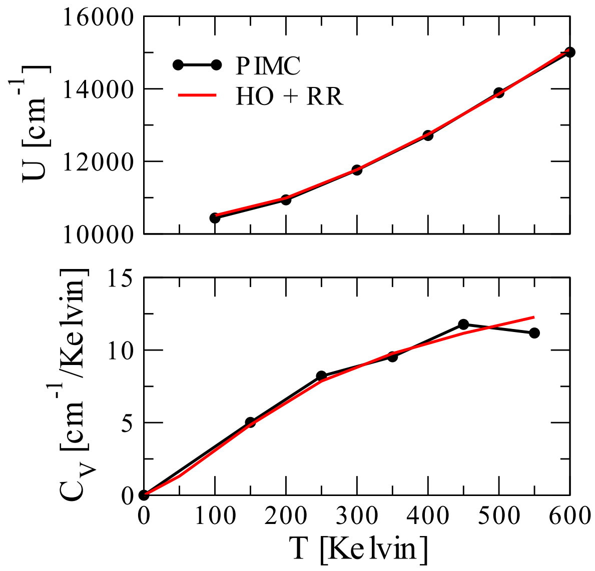

Figure S3. The ro-vibrational enthalpy *U* and specific heat C_V as functions of temperature for the highest level of path integration QM5 calculated from four independent Monte Carlo simulations with 10^5 equilibrated cycles (black curves with dots), as well as their analytic counterparts for the harmonic oscillator and rigid rotor (HO + RR, red curves). C_V was calculated by numerically differentiating the enthalpy. The value of $C_V = 0$ at 0 Kelvin is the result of the Nernst theorem.[4]

S-4. Visual analysis of the ring evolution with temperature

For a visual inspection complementary to the numerical RDF and the X-coordinate analysis, we took every 100-th structure from a single 100000 MC run (totaling 100 structures) and overlayed them onto each other in a single frame. The rotational component of the motion was removed by defining a body-fixed coordinate system (X, Y, Z) as

$$\vec{Y} = \vec{r}_1 - \vec{r}_0$$

$$\vec{Z} = (\vec{r}_4 - \vec{r}_1) \times \vec{Y}$$

$$\vec{X} = \vec{Y} \times \vec{Z}$$

followed by normalization, where $\vec{r}_j$ is the position vector of nucleus j with 0 indicating the center of mass position. We used the tools from the VMD suite[5] to make graphics. The result is shown in Figure S4 for face-up and side-on orientations.

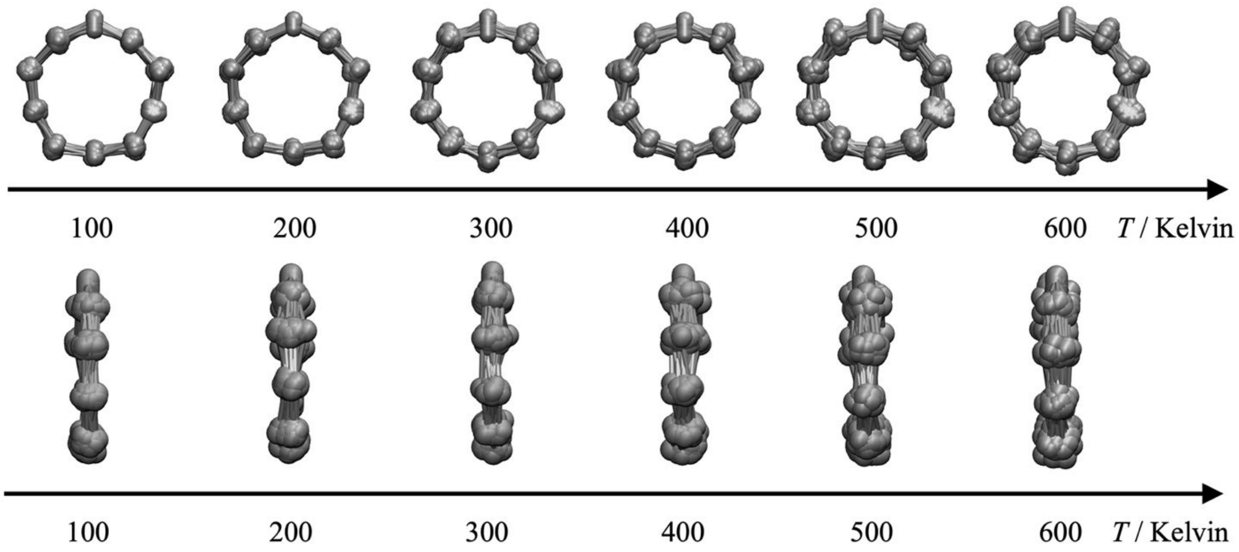

Figure S4. Vibrationally averaged structures of the cyclo[10]carbon taken from the PIMC simulations at the QM5 level at the six temperatures considered in the present work.

S-5. Calculation of the average deviation from planarity

From the definition of a plane in a 3-D Cartesian space, i.e.,

$$Ax + By + Cz + D = 0 \qquad (S6)$$

the squared distance from a point (x_i, y_i, z_i) to the plane is

$$d_i^2 = \frac{(Ax_i + By_i + Cz_i + D)^2}{A^2 + B^2 + C^2} \qquad (S7)$$

From the above it is clear that if the point (x_i, y_i, z_i) satisfies Eq. S6, it is located in the plane ($d_i = 0$); all other points lie outside the plane. For a number of points (here the nuclei of C_{10}) with i = 1, 2, ... , N = 10, we wish to minimize the average sum of the squared distances in an unconstrained manner to estimate the ring's deviation from planarity at a particular nuclear configuration m,

$$\lambda_m^2 = \min_{\{A,B,C,D\}} \frac{1}{N} \sum_{i=1}^{N} d_{i,m}^2 \qquad (S8)$$

In the present case of PIMD, the index m is chosen to represent a single "bead" (path slice) of a path integral of the Monte Carlo ensemble. The non-linear function in Eq. S8 was minimized at each configuration m in the space of the coefficients A, B, C and D using a Newton-Raphson algorithm.[6] The average over the Monte Carlo ensemble of length M is

$$\langle \lambda \rangle \equiv \left(\frac{1}{M} \sum_{m=1}^{M} \lambda_m^2 \right)^{\frac{1}{2}} \qquad (S9)$$

thus giving the deviation from planarity of the cyclo[10]carbon in a given simulation.

S-6. DVR calculations in 1-D

We estimate density shape at high temperatures by calculating the wave functions and energies in 1-D using a Fourier grid DVR.[7] To this end, a rectilinear coordinate X1, different from a non-linear coordinate X, is defined as a Cartesian vector connecting D_{10h} and D_{5h} equilibrium geometries and square-root-mass scaled by carbon atom mass in the atomic units. We then diagonalize a small DVR matrix and calculate the Boltzmann-averaged densities.

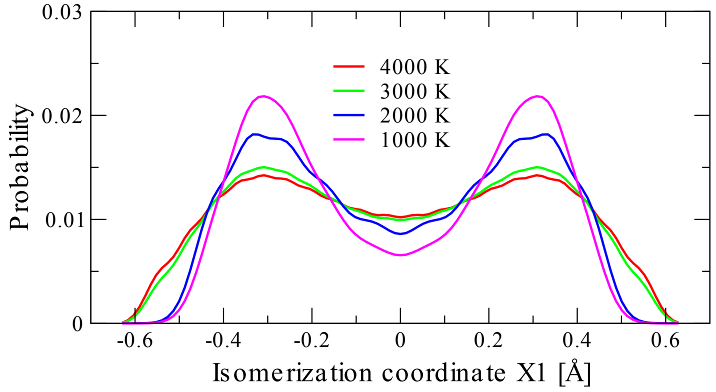

Figure S5. The DVR calculated probability distributions at high temperatures along the linear D_{5h} - D_{10h} - D_{5h} Cartesian isomerization coordinate X1. The DVR box is defined on [-0.642, 0.642] Å. The high-temperature limit flattens the density to a top hat shape.

S-7. References

[1] Gaussian 09, Revision D.01, Frisch, M. J.; Trucks, G. W.; Schlegel, H. B.; Scuseria, G. E.; Robb, M. A.; Cheeseman, J. R.; Scalmani, G.; Barone, V.; Mennucci, B.; Petersson, G. A.; Nakatsuji, H.; Caricato, M.; Li, X.; Hratchian, H. P.; Izmaylov, A. F.; Bloino, J.; Zheng, G.; Sonnenberg, J.L.; Hada, M.; Ehara, M.; Toyota, K.; Fukuda, R.; Hasegawa, J.; Ishida, M.; Nakajima, T.; Honda, Y.; Kitao, O.; Nakai, H.; Vreven, T.; Montgomery, Jr., J. A.; Peralta, J. E.; Ogliaro, F.; Bearpark, M.; Heyd, J. J.; Brothers, E.; Kudin, K.N.; Staroverov, V. N.; Keith, T.; Kobayashi, R.; Normand, J.; Raghavachari, K.; Rendell, A.; Burant, J. C.; Iyengar, S. S.; Tomasi, J.; Cossi, M.; Rega, N.; Millam, J. M.; Klene, M.; Knox, J. E.; Cross, J. B.; Bakken, V.; Adamo, C.; Jaramillo, J.; Gomperts, R.; Stratmann, R. E.; Yazyev, O.; Austin, A. J.; Cammi, R.; Pomelli, C.; Ochterski, J. W.; Martin, R. L.; Morokuma, K.; Zakrzewski,V. G.; Voth, G.A.; Salvador, P.; Dannenberg, J. J.; Dapprich, S.; Daniels, A.D.; Farkas, O.; Foresman, J. B.; Ortiz, J. V.; Cioslowski, J. and Fox, D. J.; Gaussian, Inc., Wallingford CT, 2013.

[2] Hamill, L. A.; Snyder, J. D.; Ess, D. H. (2016) MECPro Version 1.0.3: Minimum Energy Crossing Program.

[3] Janke, W.; Sauer, T. Optimal energy estimation in path-integral Monte Carlo simulations. *J. Chem. Phys.* **1997**, *107*, 5821-5839.

[4] McQuarrie, D. A. *Statistical Mechanics*; Harper & Row: New York, 1976.

[5] Humphrey, W.; Dalke, A.; Schulten, K. VMD - Visual Molecular Dynamics. *J. Molec. Graphics* **1996**, *14*, 33-38.

[6] Liu, D.; Nocedal, J. On the Limited Memory BFGS Method for Large Scale Optimization. *Mathematical Programming B* **1989**, *45*, 503-528.

[7] Colbert, D. T.; Miller, W. H. A novel discrete variable representation for quantum mechanical reactive scattering via the S-matrix Kohn method. *J. Chem. Phys.* **1991**, *96*, 1982-1991.
